# Naphtha in Phthisis

**Published:** 1844-05-04

**Authors:** 


NAPHTHA IN PHTHISIS.

Dr. May, of Maldon, narrates several cases of phthisis, in which naphtha entirely failed. Like all vaunted specifics, this remedy (?) seems to have had its day, and to have gained a loss of reputation (Hibernice) sooner than any other. It will soon be entirely forgotten.—*Medical Times.*

